# Applications of large language models in cancer care: current evidence and future perspectives

**DOI:** 10.3389/fonc.2023.1268915

**Published:** 2023-09-04

**Authors:** Giovanni Maria Iannantuono, Dara Bracken-Clarke, Charalampos S. Floudas, Mario Roselli, James L. Gulley, Fatima Karzai

**Affiliations:** ^1^ Genitourinary Malignancies Branch, Center for Cancer Research, National Cancer Institute, National Institutes of Health, Bethesda, MD, United States; ^2^ Medical Oncology Unit, Department of Systems Medicine, University of Rome Tor Vergata, Rome, Italy; ^3^ Center for Immuno-Oncology, Center for Cancer Research, National Cancer Institute, National Institutes of Health, Bethesda, MD, United States

**Keywords:** artificial intelligence, large language models, chatbot, cancer care, ChatGPT

## Abstract

The development of large language models (LLMs) is a recent success in the field of generative artificial intelligence (AI). They are computer models able to perform a wide range of natural language processing tasks, including content generation, question answering, or language translation. In recent months, a growing number of studies aimed to assess their potential applications in the field of medicine, including cancer care. In this mini review, we described the present published evidence for using LLMs in oncology. All the available studies assessed ChatGPT, an advanced language model developed by OpenAI, alone or compared to other LLMs, such as Google Bard, Chatsonic, and Perplexity. Although ChatGPT could provide adequate information on the screening or the management of specific solid tumors, it also demonstrated a significant error rate and a tendency toward providing obsolete data. Therefore, an accurate, expert-driven verification process remains mandatory to avoid the potential for misinformation and incorrect evidence. Overall, although this new generative AI-based technology has the potential to revolutionize the field of medicine, including that of cancer care, it will be necessary to develop rules to guide the application of these tools to maximize benefits and minimize risks.

## 1 Introduction

Over the last decades, efforts have been made to leverage the potential of artificial intelligence (AI) in the field of medicine and healthcare (1). Artificial intelligence is the discipline of computer science aiming to build intelligent entities. It was initiated in the 1950s, contemporaneously with the development of computer science (2). Subfields of AI include knowledge representation, machine learning (ML), and natural language processing (NLP). Machine learning entails the use of algorithms to analyze large quantities of data and learn from them, aiming to use what it has learned to make informed decisions on new data (3). Deep learning (DL) has been developed recently as a set of methods based on artificial neural networks (ANN), a category of ML algorithms. DL organizes ANNs in multiple connected layers, which are able to learn and finalize decisions independently (3). Natural language processing uses computational linguistics and ML algorithms, such as DL, to enable computers to understand text in the way humans do (4).

Thus far, one of the most representative examples of AI-based technologies applied in medicine and oncology are chatbots (also known as chatter robots, smart bots, or digital assistants) (5). These are computer programs that use NLP to simulate human conversations. They are distinguished by ease of use and a straightforward general architecture based on four main stages (input processing and comprehension, followed by response generation and selection) (5, 6). After entering a query in natural language (known as a “prompt”), the chatbot replies with a natural-language response. This exchange of prompts and responses establishes the beginning of a “session” and the overall effect mimics a natural human conversation (6). Chatbots are versatile tools, as demonstrated by their broad application in oncology, including remote patient monitoring, emotional support, general lifestyle coaching, and physician treatment planning (5, 6).

In recent years, the application of DL to NLP has led to breakthroughs in the field of generative AI, as evidenced by the advent of large language models (LLMs). Generative AI includes technologies capable of creating new content in response to prompts such as text, images, or other media (7). Large language models are DL computer models with a vast quantity of parameters and trained on highly extensive datasets with unlabeled text. They can recognize, summarize, and generate new content by leveraging statistical associations between letters and words (8). Large language models can also be considered “few-shot learners” since once trained, they can adapt to new domains with a small number of examples (9). In the last year, great attention has been captured by ChatGPT, a new generation chatbot (Chat) developed based on a family of LLMs called Generative Pre-trained Transformer (GPT) (10). Beyond ChatGPT, other new-generation chatbots based on different LLMs have been recently released, including Google BARD (11), Perplexity (12), and Anthropic Claude (13).

The remarkable versatility and potential applicability of LLMs to a wide range of tasks were immediately evident following their introduction. Consequently, many studies have aimed to assess the potential role of these new-AI technologies in several fields, including medicine and healthcare (14). In addition, growing evidence for its use in oncology has become apparent, as demonstrated by an increasing number of published commentaries, editorials, and letters (15, 16). Therefore, we aimed to describe the published applications and potential impact of LLMs.

## 2 Methods

In this narrative mini review, we summarized the presently available evidence in the biomedical scientific literature for the application of LLMs to oncology. We searched PubMed for relevant articles from its inception to July 12, 2023, without applied filters. The search terms used for the literature search were discussed and established *a priori* by the authors. A two-stage study selection process (titles and abstracts screening followed by full-texts assessment) was used in the literature search. The eligibility criteria included studies assessing the role of LLMs in cancer care (both solid and hematological tumors). We first reported the findings of the literature search and, then, our perspectives on the benefits, risks, and potential future applications for generative AI-based technology.

## 3 Results

We found twelve publications assessing the potential of LLMs in oncology (**Table 1**). All the studies evaluated ChatGPT alone or in comparison with other LLMs. All the studies evaluated ChatGPT alone or in comparison with other LLMs. No studies assessing LLMs other than ChatGPT alone were found. Overall, several domains in cancer care were investigated, including both surgical and medical care of patients affected by solid tumors. No studies involving patients with hematological malignancies were found.

**Table 1:** List of studies evaluating the role of LLMs in cancer care.

First Author	Year of publication	Large Language Model		Domain	Questions (n)	Reviewers (n)
		*ChatGPT*	*Other*			
Johnson SB (17)	2023	Free version	–	“Common Cancer Myths and Misconceptions” (NCI web page)	13	5
Yeo YH (18)	2023	Free version	–	Cirrhosis or HCC	164	2
Cao JJ (19)	2023	Free version	–	HCC diagnosis and surveillance	20	6
Haver HL (20)	2023	Free version	–	Breast cancer prevention and screening	25	3
Moazzam Z (21)	2023	Free version	–	Pancreatic cancer surgical care	30	20
Coskun B (22)	2023	Free version	–	Prostate cancer diagnosis and treatment	59	2
Zhu L (23)	2023	Free and paid versions	YouChat, NeevaAI, Perplexity, Chatsonic	Prostate cancer diagnosis and treatment	22	3
Rahsepar AA (24)	2023	Free version	Google Bard	Lung cancer prevention and screening	40	2
Sorin V (25)	2023	Free version	–	Breast cancer clinical cases	10	2
Schulte B (26)	2023	Free version	–	Systemic therapies for advanced solid tumors	51	NA
Haemmerly J (27)	2023	Free version	–	Brain cancer clinical cases	10	5
O’Hern K (28)	2023	Free version	–	Common and rare cutaneous cancer clinical cases		NA

HCC, Hepatocellular carcinoma; n, Number; NA, Not Available; NCI, National Cancer Institute.

### 3.1 LLMs as “virtual assistants” in providing information about cancer

Most of the published studies aimed to investigate the ability of LLMs to provide information by answering questions related to a specific field with overall similar methodologies across studies. In the most frequent paradigm, a group of questions on a specific topic was created and submitted to one or more LLMs, with some studies including questions of varying difficulty. The resulting output was then assessed by a group of reviewers, and the responses were rated. Based on these ratings, the competence of the LLMs to provide relevant and accurate information was determined.

The first study assessing the accuracy of an LLM in answering oncologic queries was published by Johnson et al. in December 2022 (17). Five expert reviewers evaluated the responses provided by ChatGPT to 13 questions derived from the “Common Cancer Myths and Misconceptions” webpage and compared the results with those provided by the National Cancer Institute. Despite some differences in terms of readability and word count, ChatGPT provided generally accurate information on this topic (17). Subsequently, Yeo et al. assessed the ability of ChatGPT to answer questions regarding the management and emotional support for patients affected by cirrhosis and hepatocellular carcinoma (HCC) (18). Two reviewers assessed responses to 73 questions on HCC. Overall, almost three-quarters of the answers were considered correct. However, while a higher rate of accurate responses was found in the categories “basic knowledge”, “treatment”, and “lifestyle”, more than half of the answers in the “diagnosis” category contained outdated or incorrect information. Furthermore, one-third of the response in this category were defined as incorrect (18). Cao et al. reported the results of a study assessing the ability of ChatGPT to provide information on the diagnosis and surveillance of liver cancer. Twenty questions were submitted to ChatGPT, and the responses were assessed by six reviewers. Overall, these results demonstrated the poor performance of ChatGPT in providing information on liver cancer surveillance and radiological diagnosis (19). Haver et al. published the results of a retrospective study aiming to assess the appropriateness of ChatGPT in providing recommendations for breast cancer prevention and screening. Twenty-five questions were submitted to ChatGPT, with the majority of the answers considered appropriate by the reviewers (20). Moazzam et al. reported the results of an observational study assessing the performance of ChatGPT in answering 30 questions on surgical care in pancreatic care. The responses were evaluated by 30 reviewers and demonstrated the feasibility of ChatGPT in providing high-quality responses in this domain (21). Coskun et al. reported the results of a study assessing the performance of ChatGTP in providing information about prostate cancer. Fifty-nine queries were derived from the European Association of Urology patient information platform and assessed by two reviewers. The results showed that the accuracy and quality of the content generated by ChatGPT were not optimal (22).

In light of these data, the comparison of answers provided by ChatGPT with other LLMs yielded interesting results. Zhu et al. reported the results of a study assessing the feasibility of five LLMs (ChatGPT [Free and Plus version], NeevaAI, YouChat, Perplexity, and Chatsonic) for the provision of healthcare information for prostate cancer patients (23). Twenty-two questions regarding screening, diagnosis, and treatment options for prostate cancer were designed and submitted to the aforementioned LLMs. Questions were subdivided according to the difficulty into “basic” and “hard”. ChatGPT had the highest accuracy rate among the evaluated LLMs, with the free version proving superior to the paid version (23). In addition, Rahsepar et al. recently published the results of a study assessing the accuracy and consistency of responses generated by ChatGPT, Google Bard, Bing, and Google search engines on the topic of lung cancer screening and prevention with responses assessed by two radiologists. Despite the greater accuracy of ChatGPT, none of the LLMs or search engines were able to achieve a 100% correct response rate (24).

### 3.2 LLMs as “virtual assistants” in making clinical decisions

Although the majority of the available studies assessed the potential of ChatGPT to answer questions on a specific topic, initial studies evaluated ChatGPT as a treatment decision aid in various clinical case scenarios.

Sorin et al. reported the results of a retrospective study assessing ChatGPT’s ability to support the clinical decisions of a breast tumor board (25). Here, ten consecutive real-world cases of women diagnosed with breast cancer were submitted to ChatGPT. The responses thus obtained from ChatGPT were similar to those issued by the tumor board in 70% of cases. Although ChatGPT’s performance in summarizing these cases and explaining its conclusions was highly rated, the clinical decisions were not always in concordance with those provided by the tumor board (25). Another observational study demonstrated the feasibility of ChatGPT for providing guideline-based treatment decisions in solid-organ oncology cases. Here, over 50 prompts regarding 32 separate solid cancers were submitted, and the outputs were evaluated via the valid therapy quotient, a surrogate for the fraction of acceptable recommendations. In all cases, ChatGPT identified at least one treatment option suggested by National Comprehensive Cancer Network guidelines (26). Recently, Haemmerli et al. described the performance of ChatGPT in providing clinical decisions on adjuvant treatment in patients with gliomas. Ten clinical scenarios derived from an equal number of patients were submitted to ChatGPT, and the responses were reviewed by five experts. The results demonstrated that ChatGPT performed poorly in classifying glioma types but was more effective in providing adjuvant treatment recommendations (27). Finally, O’Hern et al. assessed the performance of ChatGPT in providing appropriate recommendations regarding the surgical management of cutaneous neoplasms. Thirty clinical scenarios related to common and rare cutaneous tumors were submitted to ChatGPT to determine whether wide local excision or Mohs surgery were appropriate treatment options (28).

## 4 Discussion

**Figure 1:**
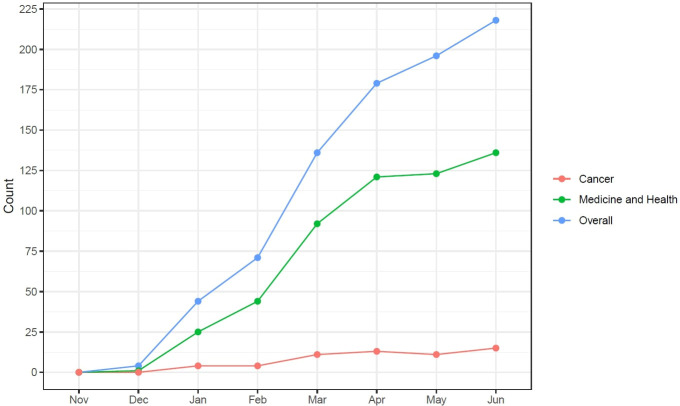
Number of publications indexed in PubMed about ChatGPT since its release.

Since the term AI was coined in 1956, the field of medicine has attempted to leverage its potential to improve patients’ lives and streamline physician workflow (2). Despite initial disappointments, recent improvements in computing power, storage, and speed, have set the stage for a revolution in the application of AI in healthcare (29). In parallel, the development of superior algorithms, better able to analyze large volumes of data, has finally made feasible the meaningful implementation of AI in medicine (6). Thus far, several AI-aided tasks have become a reality in many domains of medicine and health care, including coding medical notes, interpreting electrocardiograms, analyzing medical images, identifying high-risk patients, and detecting drug interactions (1). In this context, the recent breakthroughs observed in generative AI technology with the advent of LLMs have provided new perspectives for AI applications in medicine and healthcare. Although not specifically trained for addressing healthcare issues, the potential applications of LLMs to this domain were immediately apparent. In particular, the “cultural sensation” resulting from the remarkable versatility of ChatGPT has captured the scientific community’s attention (30). The impressive and rapidly increasing number of publications indexed in PubMed providing data or perspectives about the possible applications of this new generative AI tool is strong proof (**Figure 1**).

Therefore, we aimed to summarize the available evidence about the applications of LLMs in cancer care. Our results demonstrate that most published studies assessed the potential of ChatGPT as a “virtual assistant” for patients or physicians and were characterized by a similar methodology. They predominantly evaluate the potential for ChatGPT, alone or in comparison with other LLMs, to create novel content to answer patient and caregiver queries, with a smaller subset assessing its role as a treatment decision aid. Overall, the available data demonstrate ChatGPT as generally accurate when addressing cancer queries – albeit with a consequential error rate. The most recent studies, in contrast, compared ChatGPT with other LLMs, providing relevant information on the accuracy of ChatGPT in comparison to other LLMs. These data are likely to become more relevant over time, given both the potential increasing role of LLMs in healthcare and the proliferation of different models.

Despite the excitement generated by the potential applications of these new AI tools, some ethical implications related to their use deserve attention. Firstly, the risk of misinformation and distortion of scientific facts is evident and must not be underrated. The potential for generating medical disinformation is highly dangerous and must be addressed by the scientific community (31). As demonstrated by our results, the accuracy rate of ChatGPT and other LLMs in the field of cancer care is far from 100%. Therefore, subsequent verification of the outputs is presently mandatory in order to avoid misinterpretation, especially when dealing with biomedical data. Secondly, the data used to train ChatGPT and other major LLM are not publicly available, and thus, accurate validation of the information used to produce these outputs is not presently possible (10). Additionally, the data used to train ChatGPT is not updated in real-time and, thus, is highly prone to obsolescence – especially in a field as rapidly advancing as oncology (10). Initial evidence suggests that the behavior of the same LLM can also change over time, highlighting the need to monitor their quality continuously (32). A potential future paradigm may be represented by open-source LLMs trained with specific datasets in order to accomplish pre-specified tasks. An illustrative example of this perspective is represented by BioGPT, a cutting-edge LLM created for the biomedical field with a user-friendly interface. BioGPT was built using the same architecture as OpenAI’s GPT models but was trained using datasets derived from biomedical literature and, thus, tailored for specific tasks in the biomedical domain (33).

In parallel, several studies assessed the potential for ChatGPT to impact healthcare processes, and, despite not being specifically related to oncology, they merit discussion. ChatGPT’s abilities in summarizing and generating text may be successfully used to streamline some of the more purely bureaucratic tasks which threaten to overwhelm physicians. Initial evidence strongly suggests a role for ChatGPT in generating letters of medical necessity or discharge summaries (34). Such automation may greatly facilitate increased direct patient-physician interaction and, thus, quality of care. Notably, this is a focus of healthcare informatics, with significant effort presently ongoing to implement this technology in electronic health records. Additionally, the strengths of LLMs in pattern recognition and correlation analysis have obvious implications for applications to biomedical research and the extraction of “real-world” data for clinical research (35).

Overall, these data highlight the potential for generative AI and LLMs to impact medicine positively. Although these tools have not been specifically designed for healthcare purposes, their generalizability and the breadth of their training datasets have already rendered them remarkably powerful in the biomedical field. Thus, further assessment of its applicability to medicine in general and oncology is strongly warranted; insights gained may facilitate the subsequent development of more powerful LLMs designed for healthcare. However, important issues remain, and caution, along with expert assessment and, initially at least, review of the output, is warranted prior to being brought into general use. The potential for generating misinformation remains and must be further analyzed. Given the potential benefits of this technology and its current informal use in the biomedical and healthcare fields, it is incumbent upon the scientific community to formally and scientifically assess safety and applicability. It is evident that Pandora’s box has been opened, and potential applications of this technology are likely to multiply at an accelerating rate.

## 5 Conclusion

This review showed the potential application of LLMs in oncology, especially as “virtual assistants” for both patients and physicians. Available evidence strongly focused on assessing the potential of ChatGPT that, despite being capable of providing adequate information on specific cancer types and in certain situations, also demonstrated a significant error rate and a tendency towards providing obsolete data. Therefore, an accurate, expert-driven verification process remains mandatory to avoid the potential for misinformation and incorrect evidence. It will be essential in the near future to incorporate this new technology, given its potential to revolutionize how medicine is practiced. However, it will also be necessary to develop rules to guide the application of LLMs while maximizing benefits and minimizing risks and harms to providers and, most importantly, patients.

## Author contributions

GI: Conceptualization, Investigation, Methodology, Visualization, Writing – original draft. DB: Investigation, Writing – original draft. CF: Conceptualization, Visualization, Writing – review & editing. MR: Writing – review and editing. JG: Writing – review and editing. FK: Conceptualization, Supervision, Writing – review and editing.
